# A selective cytotoxic adenovirus vector for concentration of pluripotent stem cells in human pluripotent stem cell-derived neural progenitor cells

**DOI:** 10.1038/s41598-021-90928-7

**Published:** 2021-06-01

**Authors:** Takamasa Hirai, Ken Kono, Rumi Sawada, Takuya Kuroda, Satoshi Yasuda, Satoko Matsuyama, Akifumi Matsuyama, Naoya Koizumi, Naoki Utoguchi, Hiroyuki Mizuguchi, Yoji Sato

**Affiliations:** 1 Division of Cell-Based Therapeutic Products, National Institute of Health Sciences, 3-25-26 Tonomachi, Kawasaki Ward, Kawasaki City, Kanagawa 210-9501 Japan; 2 Department of Quality Assurance Science for Pharmaceuticals, Graduate School of Pharmaceutical Sciences, Nagoya City University, Aichi, Japan; 3 Center for Reverse TR, Osaka Habikino Medical Center, Osaka Prefectural Hospital Organization, Osaka, Japan; 4 Department of Pharmaceutics and Biopharmaceutics, Showa Pharmaceutical University, Tokyo, Japan; 5 Laboratory of Biochemistry and Molecular Biology, Graduate School of Pharmaceutical Sciences, Osaka University, Osaka, Japan; 6 LiSE Laboratory, Kanagawa Institute of Industrial Science and Technology, Kanagawa, Japan; 7 Department of Translational Pharmaceutical Sciences, Graduate School of Pharmaceutical Sciences, Kyushu University, Fukuoka, Japan; 8 Department of Cellular and Gene Therapy Products, Graduate School of Pharmaceutical Sciences, Osaka University, Osaka, Japan

**Keywords:** Biological techniques, Stem cells

## Abstract

Highly sensitive detection of residual undifferentiated pluripotent stem cells is essential for the quality and safety of cell-processed therapeutic products derived from human induced pluripotent stem cells (hiPSCs). We previously reported the generation of an adenovirus (Ad) vector and adeno-associated virus vectors that possess a suicide gene, inducible Caspase 9 (iCasp9), which makes it possible to sensitively detect undifferentiated hiPSCs in cultures of hiPSC-derived cardiomyocytes. In this study, we investigated whether these vectors also allow for detection of undifferentiated hiPSCs in preparations of hiPSC-derived neural progenitor cells (hiPSC-NPCs), which have been expected to treat neurological disorders. To detect undifferentiated hiPSCs, the expression of pluripotent stem cell markers was determined by immunostaining and flow cytometry. Using immortalized NPCs as a model, the Ad vector was identified to be the most efficient among the vectors tested in detecting undifferentiated hiPSCs. Moreover, we found that the Ad vector killed most hiPSC-NPCs in an iCasp9-dependent manner, enabling flow cytometry to detect undifferentiated hiPSCs intermingled at a lower concentration (0.002%) than reported previously (0.1%). These data indicate that the Ad vector selectively eliminates hiPSC-NPCs, thus allowing for sensitive detection of hiPSCs. This cytotoxic viral vector could contribute to ensuring the quality and safety of hiPSCs-NPCs for therapeutic use.

## Introduction

A variety of cell-processed therapeutic products (CTPs) derived from human induced pluripotent stem cells (hiPSCs) are currently under development for the treatment of life-threatening or incurable diseases. Neural progenitor cells (NPCs) derived from hiPSCs (hiPSC-NPCs) are promising candidates as a cell-based therapy for neurological disorders, such as spinal cord injury (SCI) and stroke. Several research groups have reported that hiPSC-NPCs can differentiate into neurons able to promote axonal regrowth and angiogenesis in several animal disease models, resulting in functional recovery^1–3^. Furthermore, a clinical study to treat in SCI patients is expected to start in the near future^4^. However, there are several safety issues in using hiPSC-derived CTPs as therapeutics. One of the major issues is the potential for the formation of tumors derived from the engrafted cells. Several studies have reported neural tumor-like proliferations of hiPSC-NPCs transplanted in immunodeficient rodents^5–7^. Therefore, the assessment of tumorigenicity is critical for the clinical use of hiPSC-derived CTPs, including NPCs.

Since hiPSCs possess tumorigenic potential, residual undifferentiated hiPSCs is one of the hazards for the potential risk of tumor formation from hiPSC-derived CTPs^8,9^. To date, we have developed several methods for detecting a trace amount of undifferentiated hiPSCs in CTPs, such as flow cytometry assay for tumor rejection antigens (TRA)-1-60^10^, droplet digital polymerase chain reaction (PCR) assays for *LIN28A*^11^, and the highly efficient culture (HEC) assay using a cell culture system optimized for hiPSCs^12^. The lowest detection limit among these methods for residual hiPSCs is 1/10^5^ (0.001%, undifferentiated hiPSCs/differentiated cells). In upcoming clinical trials to treat SCI, 2.0 × 10^6^ hiPSC-NPCs are expected to be transplanted into patients^13^; thus, undifferentiated hiPSCs present in the transplanted cells could remain undetected by the aforementioned test methods. To overcome their limitation in sensitivity, we constructed adenovirus serotype 5 (Ad) and adeno-associated virus serotype 1, 2, 5, and 6 (AAV1, AAV2, AAV5, and AAV6, respectively) vectors expressing a suicide gene, inducible Caspase 9 (iCasp9), under the control of the cytomegalovirus (CMV) promoter, which is dormant in iPSCs^14,15^. Theoretically, these vectors allow differentiated cell-selective expression of iCasp9, whose dimerization in response to AP1903 activates the apoptotic cascade^16^. We have shown that some of these vectors are highly cytotoxic to hiPSC-derived cardiomyocytes but not to hiPSCs, allowing for the concentration and sensitive detection of hiPSCs intermingled in cultures of hiPSC-derived cardiomyocytes^16^. These cytotoxic viral vectors are therefore promising biological tools for verifying the quality of hiPSC-derived CTPs. However, the usefulness of these viral vectors in the quality assessment of cells other than cardiomyocytes remains unclear. In this study, we investigated whether these vectors are applicable to the concentration and sensitive detection of hiPSCs intermingled in cultures of hiPSC-NPCs.

## Results

### The Ad vector efficiently transduced immortalized NPCs

**Figure 1:**
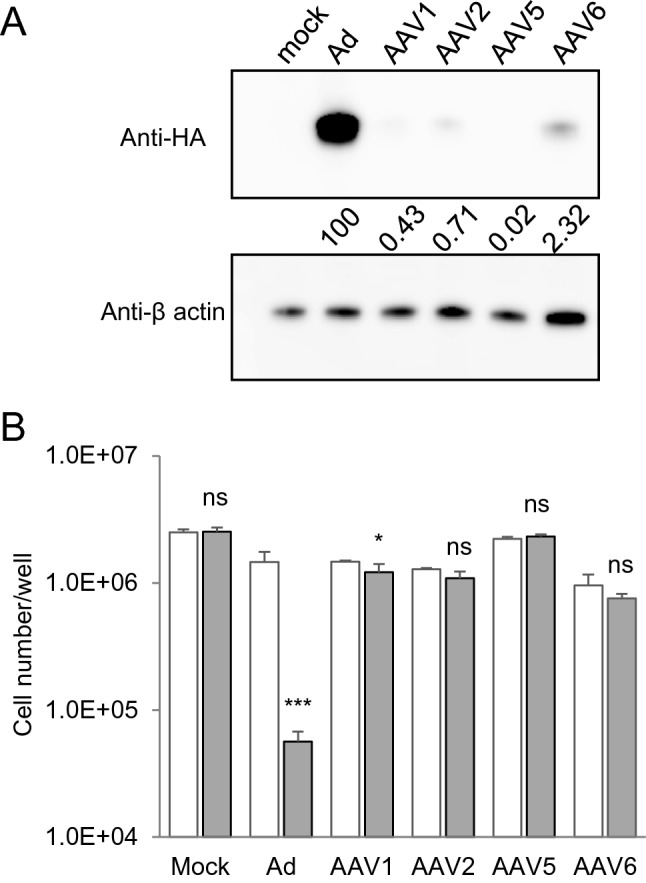
Selection of viral vectors for the transduction of NPCs. (**A**) The expression of iCasp9 with a C terminal HA-tag in lysates of immortalized NPCs transduced with the Ad vector (3 pfu/cell) and the AAV1/2/5/6 vectors (670 vg/cell) was assessed by western blotting with an antibody against the HA-tag. β-actin expression levels were assessed in parallel for normalization. The relative iCasp9 expression obtained from the band intensity of iCasp9 divided by that of β-actin is shown. Full-length blots are shown in Supplementary Fig S1. (**B**) The number of cells transduced with viral vectors was counted after treatment with or without AP1903 (open column, without AP1903; filled column, with AP1903). Data are presented as means ± standard deviation (SD) (n = 3, biological replicates). The statistical significance of differences between cells treated with and without AP1903 was determined by two-way ANOVA and SNK post-hoc test (^*^P < 0.05; ^***^P < 0.005; ns, not significant *vs.* without AP1903, SigmaPlot Version 12.5).

First, we evaluated the transduction efficiency of the viral vectors we had constructed by using immortalized NPCs as a model of hiPSC-NPCs. The immortalized NPCs were transduced with the Ad vector or four AAV vectors (AAV1, AAV2, AAV5, and AAV6) for 24 h. Western blot analysis showed that the expression levels of iCasp9 at 2 days post-transduction were the highest in cells transduced with the Ad vector among the viral vectors studied (Fig. 1A and Supplementary Fig. S1). Consistent with the levels of iCasp9 expression, when the transduced cells were treated with AP1903, a reagent that induces apoptosis via the dimerization of iCasp9, the number of Ad vector-transduced cells decreased to less than 10% of the number of untreated cells (*P* < 0.005, two-way analysis of variance (ANOVA) and Student–Newman–Keuls (SNK) post-hoc test), whereas the numbers of cells transduced with the four AAV vectors were not decreased (Fig. 1B). In contrast, neither iCasp9 expression nor a decrease in cell number was found when hiPSCs were treated with the Ad vector and AP1903 (Supplementary Fig. S2). Therefore, we used the Ad vector in these experiments to transduce the iCasp9 gene into NPCs.

**Figure 2:**
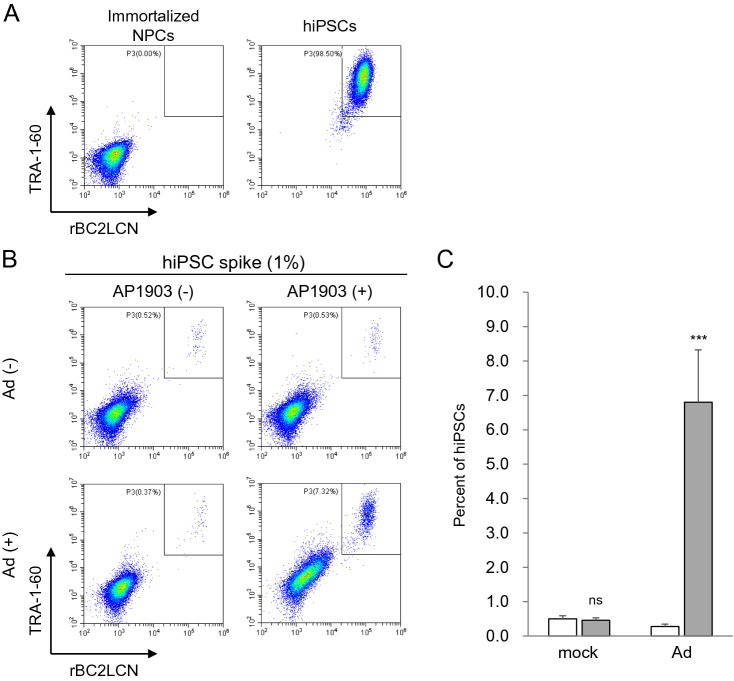
hiPSCs in cultures of immortalized NPCs are concentrated by treatment with the Ad vector and AP1903. Immortalized NPCs supplemented with hiPSCs at a ratio of 1% were treated with the Ad vector (3 pfu/cell) and AP1903. After treatment, the binding levels of rBC2LCN and the expression levels of TRA-1–60 were determined by flow cytometry. Representative pseudo color plots of the data from three independent experiments are shown (**A**, **B**). The percentages of hiPSCs in immortalized NPCs are shown as means ± SD of three independent experiments (open column, without AP1903; filled column, with AP1903) (**C**). Statistical significance was determined by two-way ANOVA and SNK post-hoc test (^***^P < 0.005; ns, not significant *vs.* without AP1903, SigmaPlot Version 12.5).

**Figure 3:**
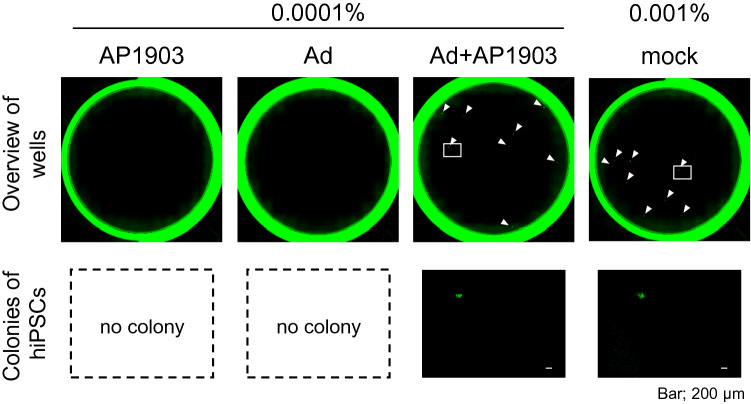
HEC assay for a trace amount of hiPSCs in cultures of immortalized NPCs after treatment with the Ad vector and AP1903. Immortalized NPCs supplemented with hiPSCs at a ratio of 0.0001% or 0.001% as a positive control were treated with the Ad vector (3 pfu/cell) and AP1903. After treatment, the cells were harvested and seeded onto laminin-521-coated 12-well plates (1–2 wells per sample). Four days after seeding, cells were immunostained with an anti-TRA-1-60 antibody. The lower panels are images, which correspond to the squares in the upper panels. The arrowheads indicate hiPSC colonies. Representative images of three independent experiments are shown. Scale bar is 200 µm.

Next, we investigated whether hiPSCs in cultures of immortalized NPCs are concentrated by the Ad vector, using flow cytometry for determining the expression levels of TRA-1-60, a marker of pluripotent stem cells, and the binding levels of rBC2LCN, a recombinant peptide corresponding to the N-terminal domain of the BC2L-C protein used as probe for hiPSCs^17^. Flow cytometry analysis showed that hiPSCs were strongly positive for TRA-1-60 and rBC2LCN, whereas immortalized NPCs were negative (Fig. 2A). When immortalized NPCs were supplemented with hiPSCs at a ratio of 1%, the percentage of hiPSCs was approximately 14-fold higher in cells treated with the Ad vector and AP1903, than in mock-treated cells (*P* < 0.005, two-way ANOVA and SNK post-hoc test) (Fig. 2B,C and Supplementary Fig. S3). In addition, we aimed to detect a trace amount of hiPSCs in cultures of immortalized NPCs, using HEC assay. Immortalized NPCs (1 × 10^7^) were supplemented with either 10 hiPSCs (0.0001%) or 100 hiPSCs as a positive control (0.001%; reported detection limit). After treatment with the Ad vector and AP1903, the surviving cells were cultured for 4 days and then immunostained for TRA-1-60. The detectable levels of hiPSCs were assessed by the formation of colonies composed of spiked hiPSCs positive for TRA-1-60. For the 0.0001% hiPSC group, colonies comprising TRA-1-60 positive cells were detected in cells treated with both the Ad vector and AP1903 in all experiments but not in cells treated with either the Ad vector or AP1903 (Fig. 3 and Table 1). The number of colonies in the 0.0001% hiPSC group treated with both the Ad vector and AP1903 was comparable to that in the untreated 0.001% hiPSC group (*P* = 0.82, one-way repeated measures ANOVA and Dunnett’s post-hoc test) (Table 1).

**Table 1:** Number of detected hiPSC colonies per well in the three independent experiments shown in Fig. 3.

hiPSC	0.0001%			0.001%
	AP1903	Ad	Ad + AP1903	Mock
Exp. 1	0	0	8	7
Exp. 2	0.5	0	3	4
Exp. 3	0	0	4	6.5
Average	0.2	0.0	5.0	5.8
S.D	0.3	0.0	2.6	1.6
*P*	0.007	0.006	0.82	–

The number of hiPSC colonies per well in the experiments is shown as means (2 wells), except for the 0.0001% hiPSC group treated with both the Ad vector and AP1903 (1 well). The average and SD were calculated from the data of three independent experiments. The *P* values were analyzed by one-way repeated measures ANOVA and Dunnett’s post-hoc test (*vs.* mock of 0.001% hiPSC, SigmaPlot Version 12.5).

### hiPSC-NPCs were efficiently transduced with the Ad vector and expressed the iCasp9 transgene under the control of the CMV promoter

We next investigated whether hiPSC-NPCs, as well as immortalized NPCs, could be transduced with the Ad vector and be killed in an iCasp9-dependent manner. Fluorescence microscopy showed that PAX6, a marker of NPCs, was not expressed in hiPSCs, but in induced cells, which were defined in the present study as cells obtained by culturing hiPSCs in the differentiation medium for 16 days. In contrast, TRA-1-60 was not expressed in the induced cells (Supplementary Fig. S4A). Similarly, flow cytometry analysis showed that more than 95% of the induced cells were PAX6 positive and TRA-1-60 negative (Supplementary Fig. S4B). Moreover, quantitative real-time PCR analysis showed that pluripotency marker genes (*LIN28A*, *OCT3/4*, *NANOG*, and *ESRG*) in the induced cells were decreased compared with the hiPSCs, whereas NPC marker genes (*PAX6*, *SOX-1*, and *NES*) were increased (Supplementary Fig. S4C). These results indicate that the induced cells consist mostly of hiPSC-NPCs and contain few hiPSCs. In addition, we confirmed that coxsackievirus and adenovirus receptor (CAR), a primary receptor of Ad serotype 5, was expressed on the cell surfaces of the induced cells, as well as the immortalized NPCs used in this study (Supplementary Fig. S5). Therefore, the induced cells are hereafter referred to as hiPSC-NPC preparations.

**Figure 4:**
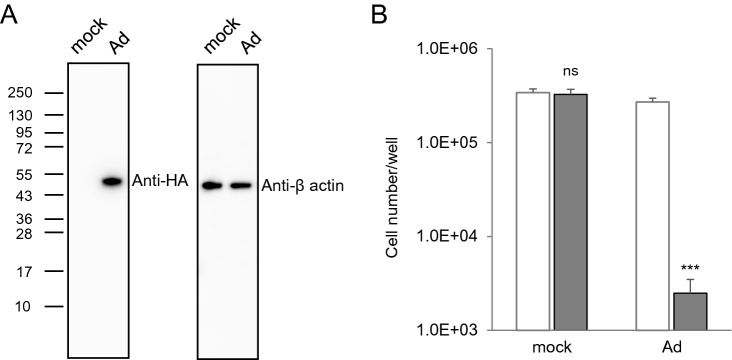
The Ad vector efficiently transduces and kills cells in hiPSC-NPC preparations in an iCasp9-dependent manner. (**A**) The expression of iCasp9 with a C terminal HA-tag in lysates of hiPSC-NPC preparations transduced with the Ad vector (18 pfu/cell) was assessed by western blotting with antibodies against HA-tag. β-actin expression levels were assessed in parallel as a loading control. (**B**) The number of living cells in hiPSC-NPC preparations transduced with the Ad vector (18 pfu/cell) was counted after treatment with or without AP1903 (open column, without AP1903; filled column, with AP1903). Data are presented as means ± SD of three independent experiments. Statistical significance was determined by two-way ANOVA and SNK post-hoc test (^***^P < 0.005; ns, not significant *vs.* without AP1903, SigmaPlot Version 12.5).

To evaluate the transduction efficiency with the Ad vector, hiPSC-NPC preparations were transduced with the Ad vector for 24 h, after which iCasp9 expression levels at 2 days post-transduction were determined by western blot analysis. As shown in Fig. 4A and Supplementary Fig. S6, the expression of iCasp9 after treatment with the Ad vector was confirmed, indicating that hiPSC-NPC preparations were efficiently transduced with the Ad vector and expressed the iCasp9 gene under the control of the CMV promoter. Moreover, the number of living cells in hiPSC-NPC preparations transduced with the Ad vector was significantly decreased to less than 10% following treatment with AP1903 (*P* < 0.005, two-way ANOVA and SNK post-hoc test) (Fig. 4B).

### Treatment with the Ad vector and AP1903 concentrated undifferentiated hiPSCs in cultures of hiPSC-NPC preparations

**Figure 5:**
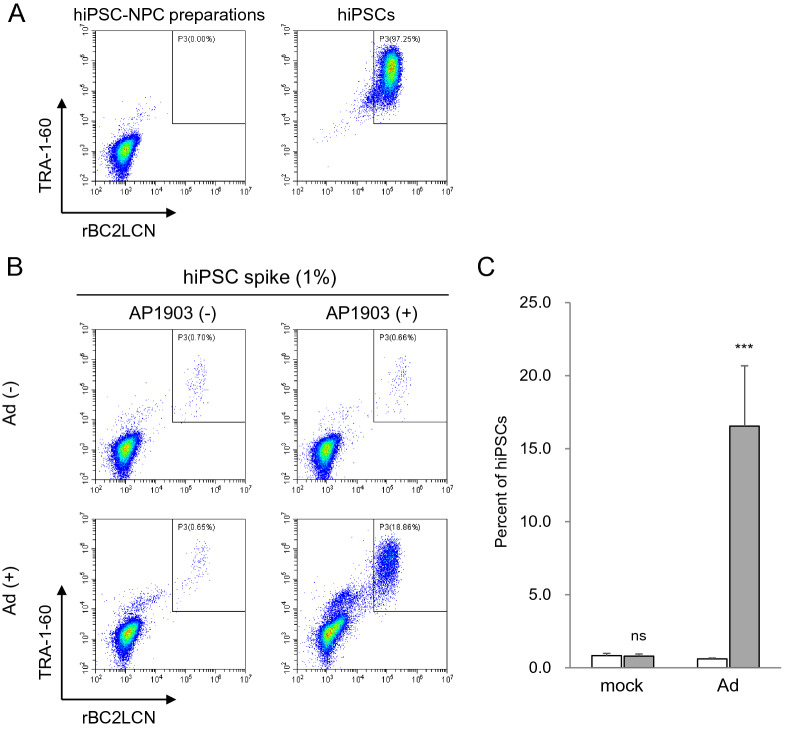
hiPSCs in cultures of hiPSC-NPC preparations are concentrated by treatment with the Ad vector and AP1903. hiPSC-NPC preparations supplemented with hiPSCs at a ratio of 1% were treated with the Ad vector (18 pfu/cell) and AP1903. After treatment, the binding levels of rBC2LCN and the expression levels of TRA-1-60 were determined by flow cytometry. Representative pseudo color plots of the data from three independent experiments are shown (**A**, **B**). The percentages of hiPSCs in culture of hiPSC-NPC preparations are shown as means ± SD of three independent experiments (open column, without AP1903; filled column, with AP1903) (**C**). Statistical significance was determined by two-way ANOVA and SNK post-hoc test (^***^P < 0.005; ns, not significant *vs.* without AP1903, SigmaPlot Version 12.5).

**Figure 6:**
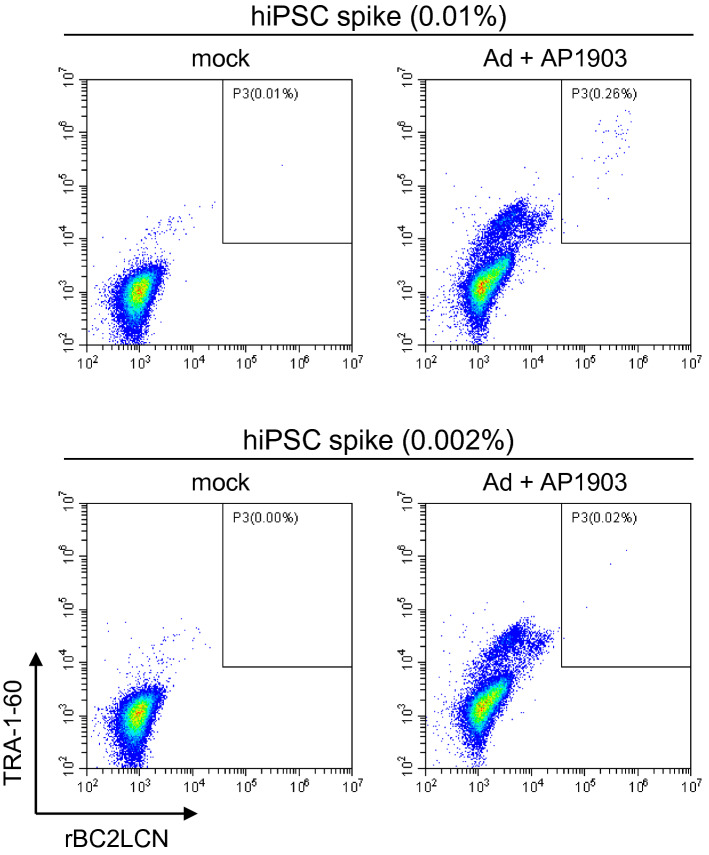
Treatment with the Ad vector and AP1903 improves the detection sensitivity for a trace amount of hiPSCs in cultures of hiPSC-NPC preparations. hiPSC-NPC preparations supplemented with hiPSCs at a ratio of 0.01% (upper panels) and 0.002% (lower panels) were treated with the Ad vector (18 pfu/cell) and AP1903. After treatment, the binding levels of rBC2LCN and the expression levels of TRA-1-60 were determined by flow cytometry. Representative pseudo color plots of the data from three independent experiments are shown.

We investigated whether treatment with the Ad vectors and AP1903 selectively eliminated hiPSC-NPCs but not undifferentiated cells, using flow cytometry. As shown in Fig. 5A and Supplementary Fig. S7, cells strongly positive for TRA-1-60 and rBC2LCN, which potentially include hiPSCs, were not detected in cultures of hiPSC-NPC preparations although cells weakly positive for both or either of them were detected. To accurately evaluate the capability of the Ad vectors to concentrate undifferentiated hiPSCs, hiPSC-NPC preparations supplemented with hiPSCs at a ratio of 1% were used. Flow cytometry analysis showed that the percentage of hiPSCs was approximately 20-fold higher in cells treated with the Ad vector and AP1903 than in mock-treated cells (*P* < 0.005, two-way ANOVA and SNK post-hoc test) (Fig. 5B,C and Supplementary Fig. S8). Furthermore, the same analyses were performed to investigate whether the Ad vector improved the detection sensitivity of flow cytometry, using hiPSC-NPC preparations supplemented with hiPSCs at ratios of 0.01 and 0.002%. We found that hiPSCs in cultures of hiPSC-NPC preparations treated with the Ad vector and AP1903 were detected, even in the 0.002% hiPSC group, but not in mock-treated cells (Fig. 6, Table 2, and Supplementary Fig. S9). The regression coefficient of the group treated with the Ad vector and AP1903 in Table 2 for the straight line passing through (0,0) i.e. the origin was 0.997 ± 0.004 (mean ± S.D., n = 3), suggesting the quantitativity of the assay at least between 0.002% and 0.01% of hiPSCs.

**Table 2:** Number of detected hiPSCs in the three independent experiments shown in Fig. 6.

hiPSC (%)	0.01		0.002	
	Mock	Ad + AP1903	Mock	Ad + AP1903
Exp. 1	2	39	0	9
Exp. 2	1	52	0	4
Exp. 3	2	57	0	11
Average	1.7	49.3	0	8.0
S.D	0.6	9.3	0	3.6
*P*	–	< 0.005	-	0.043

The number of hiPSCs per 20,000 cells in gates 1 and 2 is shown. The average and SD were calculated from the data of three independent experiments. The *P* values (one tailed) were analyzed by two-way repeated measures ANOVA and SNK post-hoc test (*vs.* mock of the same hiPSC concentration, SigmaPlot Version 12.5).

## Discussion

Reduction in the level of undifferentiated hiPSCs, as tumorigenic impurities, and their detection are necessary to ensure the quality and safety of hiPSC-derived CTPs. Proving total absence of residual hiPSCs in the products is desirable but difficult due to the limits of detection of test methods. Therefore, it is important to understand and reduce the detection limits of the test methods for evaluation of the risk and benefit balance of using the products. However, the detection limits of most methods available for the detection of residual undifferentiated hiPSCs are 1/10^5^ (0.001%) or more^9^, which might be insufficient if over 1 × 10^5^ cells are needed to treat diseases such as SCI. In this study, we investigated whether cytotoxic viral vectors constructed in our previous study^16^ can selectively eliminate differentiated cells in cultures of hiPSC-NPC preparations to enable sensitive detection of residual undifferentiated hiPSCs.

Our findings indicated that the Ad vector is the most appropriate among the vectors tested in this study to transduce the iCasp9 gene into NPCs. The expression of viral receptors on the cell surface is one of the key factors for successful transduction. Ad serotype 5 binds to CAR as a primary receptor^18,19^. Immortalized NPCs and hiPSC-NPC preparations express CAR on their surface, which contributes to high expression levels of iCasp9 in cells transduced with the Ad vector. These data show that the Ad vector can transduce NPCs with high efficiency and that the CMV promoter is active in hiPSC-NPC preparations. In contrast, the expression levels of iCasp9 in immortalized NPCs transduced with AAV vectors were low, and treatment with AP1903 did not result in a reduction in cell numbers. While AAV vectors can transduce various cell types, they are inefficient for transducing stem cell types^20–22^. The immortalized NPCs used in this study can differentiate into neurons and glial cells^23,24^. In addition, others and we have reported that AAV-mediated transgene expression is delayed in primary and hiPSC-derived cardiomyocytes and that iCasp9-dependent cytotoxicity cannot be detected at two days post-transduction^16,25^. Therefore, the low transduction efficiencies of the AAV vectors may be attributable to their long latency and the stem cell-like properties of NPCs.

In our preliminary study, we found that the higher titer of the Ad vector was needed to kill hiPSC-NPC preparations in an iCasp9-dependent manner compared with immortalized NPCs (Supplementary Fig. S10). The cytotoxicity of the Ad vector in an iCasp9-dependent manner is mainly depend on both transduction efficiency and CMV promoter activity in cells. There was no significant difference in the expression of CAR on the cell surface between immortalized NPCs and hiPSC-NPCs preparations (Supplementary Fig. S5), suggesting that entry of the Ad vector into those cells was comparable. On the other hand, CMV promoter activity is known to be dormant in undifferentiated hiPSCs and upregulated during the differentiation of hiPSCs^14–16^. Therefore, it is likely that CMV promoter activity in hiPSC-NPCs preparations was lower than immortalized NPCs, resulting in the requirement of high viral titer to abundantly express iCasp9 in almost all hiPSC-NPCs preparations.

There are various markers for pluripotent stem cells, including stage-specific embryonic antigen (SSEA)-3, SSEA-4, TRA-1-60, and TRA-1-81, which can be recognized by antibodies^26–30^, and Fucα1–2Galβ1–3GlcNAc (GalNAc)-containing glycans, which can be recognized by rBC2LCN^17,31^. Although flow cytometry analysis can detect residual undifferentiated hiPSCs in differentiated cells, by using pluripotent stem cell markers, its detection limit is reported to be 0.1%^10^. We showed that the proportion of undifferentiated hiPSCs was increased in cells treated with the Ad vector and AP1903 by about 20-fold compared with that in mock-treated cells, when hiPSC-NPC preparations were supplemented with hiPSCs at a concentration of 1%. In addition, when cells were treated with the Ad vector and AP1903, hiPSCs intermingled in hiPSC-NPC preparations at a concentration of not only 0.01% but also 0.002% were detected by flow cytometry. These results demonstrated that a trace amount of hiPSCs in cultures of NPCs was effectively enriched by treatment with the Ad vector and AP1903, thereby significantly increasing the detection sensitivity of flow cytometry analysis for undifferentiated hiPSCs.

We previously showed that hiPSCs could be transduced with Ad vector, which possesses ZsGreen gene under the control of the EF1α promoter instead of iCasp9 gene under the CMV promoter, with high efficiency (almost 100%)^16^. The hiPSCs transduced with the Ad vector containing the CMV promoter showed no expression of iCasp9 (Supplementary Fig. S2), presumably due to the dormancy of the CMV promoter in the hiPSCs. Therefore, hiPSCs intermingled in the hiPSC-NPC preparations were not eliminated by treatment with the Ad vector and AP1903, and the cytotoxicity of the Ad vector in hiPSCs is considered negligible. Moreover, we found that treatment with the Ad vector and AP1903 could concentrate hiPSCs intermingled in NPCs by 10 to 20-fold. Similarly, hiPSCs intermingled at a ratio of 0.01% were enriched in hiPSC-NPC preparations by 29-fold by the treatment with the Ad vector and AP1903. Unfortunately, it was technically difficult to produce a large and sufficient number of hiPSC-NPCs for spiking a reliable number of live hiPSCs at a ratio of less than 0.002% in order to perform HEC assays. However, the similar enrichment would be achieved, even if undifferentiated hiPSCs is less than 0.002% in hiPSC-NPC preparations.

We found that cells weakly positive for TRA-1-60 and/or rBC2LCN were present in hiPSC-NPC preparations, and we categorized cell types found in hiPSC-NPC preparations into four groups according to their marker (TRA-1-60, rBC2LCN, and PAX6) expression patterns (Supplementary Table S1). Cells weakly positive for TRA-1-60- and/or rBC2LCN and negative for PAX6 in the culture of hiPSC-NPC preparations, termed here as immature non-NPC cells, were effectively enriched by treatment with the Ad vector and AP1903, as well as hiPSCs. In addition, the proportion of TRA-1-60-, rBC2LCN-, and PAX6-negative cells, which were considered to be the population of differentiated cells other than NPCs, was increased by treatment with the Ad vector and AP1903. As the differentiation of hiPSCs into NPCs proceeds, iCasp9 expression induced by the Ad vector is expected to be upregulated via lifting of the dormancy of the CMV promoter, which leads to AP1903-dependent apoptosis. Therefore, the enrichment of the immature non-pluripotent cells and partially/fully differentiated non-NPCs after the treatment with the Ad vector and AP1903 was presumably due to their low CMV promoter activity or low transduction efficiency of viral vectors for certain differentiated cell types. This suggests that the Ad vector has the potential to concentrate non-objective cells, and further characterization of the surviving cells would provide insights into the mechanisms of not only NPC differentiation but also NPC-derived tumor formation.

On the other hand, autofluorescence or noise from dead cells/debris might lead to false-positive or false-negative by the flow cytometry analysis, which needs to be taken into consideration for accurate assessments. However, cells highly positive for both TRA-1-60 and rBC2LCN was not detected even in hiPSC-NPC preparations treated with the Ad vector and AP1903 (Supplementary Fig. S7). In addition, the population of the estimated dead cells surrounded by gate 3 shown in Supplementary Fig. S9 was negative for both TRA-1-60 and rBC2LCN. These results suggest that, even if dead cells and debris were contained in the gates 1 and 2, they were not detected as cells highly positive for both TRA-1-60 and rBC2LCN.

Besides flow cytometry, various other methods can be used for the detection of undifferentiated cells. PCR-based assays for *LIN28A* possess ability to detect a trace of hiPSCs in hiPSCs-derived products^11^. In this study, *LIN28A* was highly detected in hiPSC-NPC preparations although few cells positive for TRA-1-60 or rBC2LCN were detected in hiPSC-NPCs preparations (approximately 0.7%, Supplementary Table S1). We previously reported that, in the differentiation of hiPSCs into mesenchymal stem/stromal cells, the time courses of the reduction in the expression were not the same between the PSC marker genes, and the reduction of *LIN28* expression was slower than *OCT3/4* and *NANOG*^12^, as observed in the present study. These results suggested that the persisting expression of *LIN28* could be derived not only from the immature cellular impurities, but also hiPSC-NPCs, and that PCR-based assays for *LIN28A* was not suitable for detection of undifferentiated cells in the cultures of hiPSC-NPC preparations. The HEC assay is one of the detection methods for pluripotent stem cells and it assesses the formation of colonies composed of residual undifferentiated hiPSCs^12^. As shown in Fig. 2, hiPSCs intermingled in immortalized NPCs could proliferate and form colonies. As the cell number needed for assay increases, it is more difficult and time-consuming to find the colonies of hiPSCs. Treatment with the Ad vector and AP1903 reduces the number of NPCs, but not hiPSCs, thereby enabling cells to be seeded on fewer and/or smaller dishes, compared with no treatment. In this study, the 1 × 10^7^ immortalized NPCs seeded on 100-mm dishes could be seeded in a 12-well plate after treatment with the Ad vector and AP1903. In the HEC assay, the Ad vector would be valuable for easily and briefly finding the colonies of hiPSCs. Recently, we succeeded in improving the HEC assay to increase its sensitivity^32^. In the improved HEC assay, the enrichment step of hiPSCs by magnetic-activated cell sorting allows us to detect hiPSCs intermingled in differentiated cells at a concentration of 0.00002%, which may be superior to the enrichment method using differentiated cell-selective cytotoxic vectors. However, some other characteristics, such as operability and throughput, are likely to be quite different between the two methods. Understanding the performance, advantages, and disadvantages of each method and selecting appropriate test methods at appropriate development/manufacturing stages are critical to ensuring the quality and safety of hiPSC-derived CTPs as well as their efficient and sound scientific development.

In this study, we have successfully decreased the detection limit of the HEC assay and flow cytometry analysis for hiPSCs intermingled in NPCs by developing a method that concentrates undifferentiated cells in NPCs through a selective cytotoxic viral vector. Therefore, cytotoxic viral vectors could be a useful tool for testing the quality of various hiPSC-derived CTPs. However, the effects of cytotoxic viral vectors greatly depend on their transduction efficiencies. When using a differentiated cell-selective cytotoxic vector in a cell quality test, the vector should have a high tropism to the type of differentiated cells in the CTP. Therefore, constructing an array of cytotoxic vectors derived from a variety of types of viruses would greatly contribute to ensuring the quality and safety of various CTPs derived from human pluripotent stem cells.

## Materials and methods

### Cells and viral vectors

Immortalized human NPCs (ReNcell VM Human Neural Progenitor Cell Line; Millipore, Billerica, MA, USA) were cultured on CTS CELLstart Substrate (Thermo Fisher Scientific, Waltham, MA, USA)-coated dishes in StemPro NSC SFM (Thermo Fisher Scientific) supplemented with 200 mM GlutaMAX (Thermo Fisher Scientific), 100 U/mL penicillin, and 100 µg/mL streptomycin (Thermo Fisher Scientific). The hiPSC cell line 253G1 was obtained from the RIKEN cell bank and maintained on laminin-521 (BioLamina, Sundbyberg, Sweden) or hESC-qualified matrigel (Corning, Corning, NY, USA)-coated dishes in mTeSR1 or mTeSR Plus media (Veritas, Tokyo, Japan). For differentiation of hiPSCs into NPCs, hiPSCs were seeded onto hESC-qualified Matrigel-coated 6-well plates or 60-mm dishes and cultured in STEMdiff SMADi Neural Induction medium (Veritas) for at least 16 days according to the manufacturer’s instruction.

The constructed Ad serotype 5 vector and AAV1, 2, 5, and 6 vectors were used in this study^16^. These vectors drive the expression of a full-length iCasp9 with a hemagglutinin (HA) tag (YPYDVPDYAA) at its C-terminus under the control of the CMV promoter.

### Transduction with viral vectors and treatment with AP1903

Cells were transduced with the Ad vector at 3 or 18 pfu per cell and with the AAV vectors at 670 viral genome copies (vg) per cell for 24 h, followed by adding 10 nM AP1903 (MedChem Express, Monmouth Junction, NJ, USA) to cells. Twenty-four hours after treatment, the number of cells and protein expression levels were determined.

### Determination of cell number

After AP1903 treatment, cells were washed with phosphate-buffered saline (PBS) and harvested by treatment with Accutase (Thermo Fisher Scientific). Harvested cells were centrifuged at 450 × *g* for 5 min and then resuspended in fresh culture medium.

Aliquots of the cell suspension were stained with an Acridine Orange/Propidium Iodide Viability Kit (Logos Biosystems, Annandale, VA, USA) and quantified using a LUNA-FL Dual Fluorescence Cell Counter (Logos Biosystems).

### Western blot analysis

Cells transduced with the Ad vector and the AAV vectors were lysed in Cell Lysis Buffer M (Fujifilm Wako Pure Chemical Corporation, Osaka, Japan). Lysates were subjected to sodium dodecyl sulfate–polyacrylamide gel electrophoresis (4‒12% polyacrylamide gel) and electrophoretically transferred to a membrane (Immobilon; Millipore). Blots were blocked and probed with a high affinity anti-HA rat monoclonal antibody (Roche, Mannheim, Germany, #11,867,423,001; 1:1,000) overnight at 4 °C or with an anti-β-actin rabbit polyclonal antibody (Abcam, Cambridge, UK, #ab8227; 1:5,000) for 2 h at 15–25 °C. Blots were then incubated for 2 h at 15–25 °C either with a peroxidase-conjugated goat anti-rat IgG F(ab')_2_ fragment antibody (Jackson Lab, #112–036-062; 1:10,000) or a donkey anti-rabbit IgG F(ab')_2_ fragment antibody (Abcam, #ab98440; 1:3,000) to detect bound anti-HA or anti-actin antibodies, respectively. Bound antibodies were visualized using a Chemilumi-One Chemiluminescent Kit (Nacalai Tesque, Kyoto, Japan).

### HEC assay

hiPSCs at a concentration of 1000 cells/mL were prepared by serial dilution and 10 cells (10 µL) or 100 cells (100 µL) of hiPSCs were added to 1 × 10^7^ immortalized NPCs. The cells were seeded onto laminin-521-coated 100-mm dishes in mTeSR1 medium and transduced with the Ad vector at 3 pfu/cell for 24 h, followed by adding 10 nM AP1903. Twenty-four hours after treatment, one tenth of the untreated 0.001% hiPSC group and the 0.0001% hiPSC group treated with either the Ad vector or AP1903, and all of the 0.0001% hiPSC group treated with both the Ad vector and AP1903 were seeded onto laminin-521-coated 12-well plates. Four days after seeding, cells were fixed with 4% paraformaldehyde in PBS for 15 min, permeabilized with 0.1% Triton X-100 in PBS for 10 min, and then blocked with Blocking One (Nacalai Tesque) for 2 h at 15–25 °C. The fixed cells were then incubated with an anti-TRA-1-60 mouse monoclonal antibody (Merck, Darmstadt, Germany, #MAB4360; 1:100) for 1 h at 15–25 °C, followed by staining with a goat anti-mouse IgM conjugated with Alexa Fluor 488 (Thermo Fisher Scientific, #A21042; 1:100) for 1 h at 15–25 °C. The stained cells were visualized using a Keyence BZ-X710 All-in-one Fluorescence Microscope (Keyence, Osaka, Japan).

### Flow cytometry analysis

Immortalized NPCs (1 × 10^6^ cells) supplemented with 1 × 10^4^ hiPSCs (1%) were seeded onto hESC-qualified Matrigel-coated 6-well plates in mTeSR Plus, and hiPSC-NPC preparations (2.5 × 10^6^ cells) supplemented with 2.5 × 10^4^, 250, and 50 hiPSCs (1%, 0.01%, and 0.002%, respectively) were seeded onto hESC-qualified Matrigel-coated 60-mm dishes in STEMdiff neural progenitor medium (Veritas) with 10 µM Y27632 (Fujifilm Wako Pure Chemical Corporation). Cells were transduced with the Ad vector at 3 or 18 pfu/cell for 24 h, followed by adding 10 nM AP1903. Twenty-four hours after treatment, cells were harvested by treatment with Accutase, and the cell suspension was incubated with an anti-TRA-1-60 monoclonal antibody conjugated with DyLight 650 (Thermo Fisher Scientific, #MA1-023-D650; 1:100) and with a FITC conjugated rBC2LCN (Fujifilm Wako Pure Chemical Corporation, #186-02,993; 1:100) on ice for 1 h, followed by washing with PBS. In the experiments using hiPSC-NPC preparations, the cells were fixed using a Foxp3/Transcription Factor Staining Buffer Set (Thermo Fisher Scientific) on ice for 30 min. The stained cells were determined and analyzed using a cytoFLEX S (Beckman Coulter, Brea, CA, USA). The measurements were stopped when 2 × 10^4^ cells in the gates 1 and 2 shown in Supplementary Figs. S3, S8, and S9 were counted.

### Statistical analysis

Statistical significance was analyzed using SigmaPlot Version 12.5 (Systat Software, San Jose, CA, USA) by two-way ANOVA with SNK post-hoc test, two-way repeated measures ANOVA with SNK post-hoc test, or one-way repeated measures ANOVA with Dunnett’s post-hoc test. *P* values < 0.05 were considered significant.

## Supplementary Information

Supplementary Information.
