# Supplementary material for: A selective cytotoxic adenovirus vector for concentration of pluripotent stem cells in human pluripotent stem cell-derived neural progenitor cells
Source: Sci Rep. 2021 Jun 1;11:11407. doi: 10.1038/s41598-021-90928-7 (PMC8169681; doi:10.1038/s41598-021-90928-7)
Supplement: Supplementary file 1 — Supplementary Information. [file 41598_2021_90928_MOESM1_ESM.pdf]

A selective cytotoxic adenovirus vector for concentration of pluripotent stem cells in human pluripotent stem cell-derived neural progenitor cells

Takamasa Hirai, Ken Kono, Rumi Sawada, Takuya Kuroda, Satoshi Yasuda, Satoko Matsuyama, Akifumi Matsuyama, Naoya Koizumi, Naoki Utoguchi, Hiroyuki Mizuguchi, Yoji Sato

Supplementary methods

Quantitative real-time polymerase chain reaction.

Total RNA was isolated from hiPSCs and induced cells (hiPSC-NPC preparations) using an RNeasy Mini Kit (Qiagen) and treated with DNase I according to the manufacturer's instructions. qRT-PCR was performed with the QuantiTect Probe One-Step RT-PCR Kit (Qiagen) on a StepOnePlus Real-Time PCR System (Applied Biosystems). The expression levels of target genes were normalized to those of the GAPDH transcript, which were quantified using TaqMan human GAPDH control reagents (Thermo Fisher Scientific, # 402869). Probes and primers were obtained from Sigma-Aldrich. The sequences of the primers and probes used in the present study are listed in Supplementary Table S2.

Immunofluorescence staining.

hiPSCs and induced cells (hiPSC-NPC preparations) were fixed with 4% paraformaldehyde in PBS (Nacalai) for 30 min at 15–25 °C. After washing with PBS, the cells were permeabilized with 0.1% Triton-X100 in PBS for 10 min and blocked with Blocking One (Nacalai) for 1 h. The cells were incubated with anti-TRA-1-60 mouse monoclonal antibody (Millipore; #MAB4360, 1:100) and rabbit anti-PAX6 antibody (BioLegend; #PRB-278P-100, 1:200) for 1 h, followed by staining with goat anti-mouse IgM Alexa Fluor 488 and goat anti-rabbit IgG Alexa Fluor 555 secondary antibodies (Invitrogen) for 1 h at 15–25 °C. The samples were examined using a Keyence BZ-X710 All-in-one Fluorescence Microscope (KEYENCE).

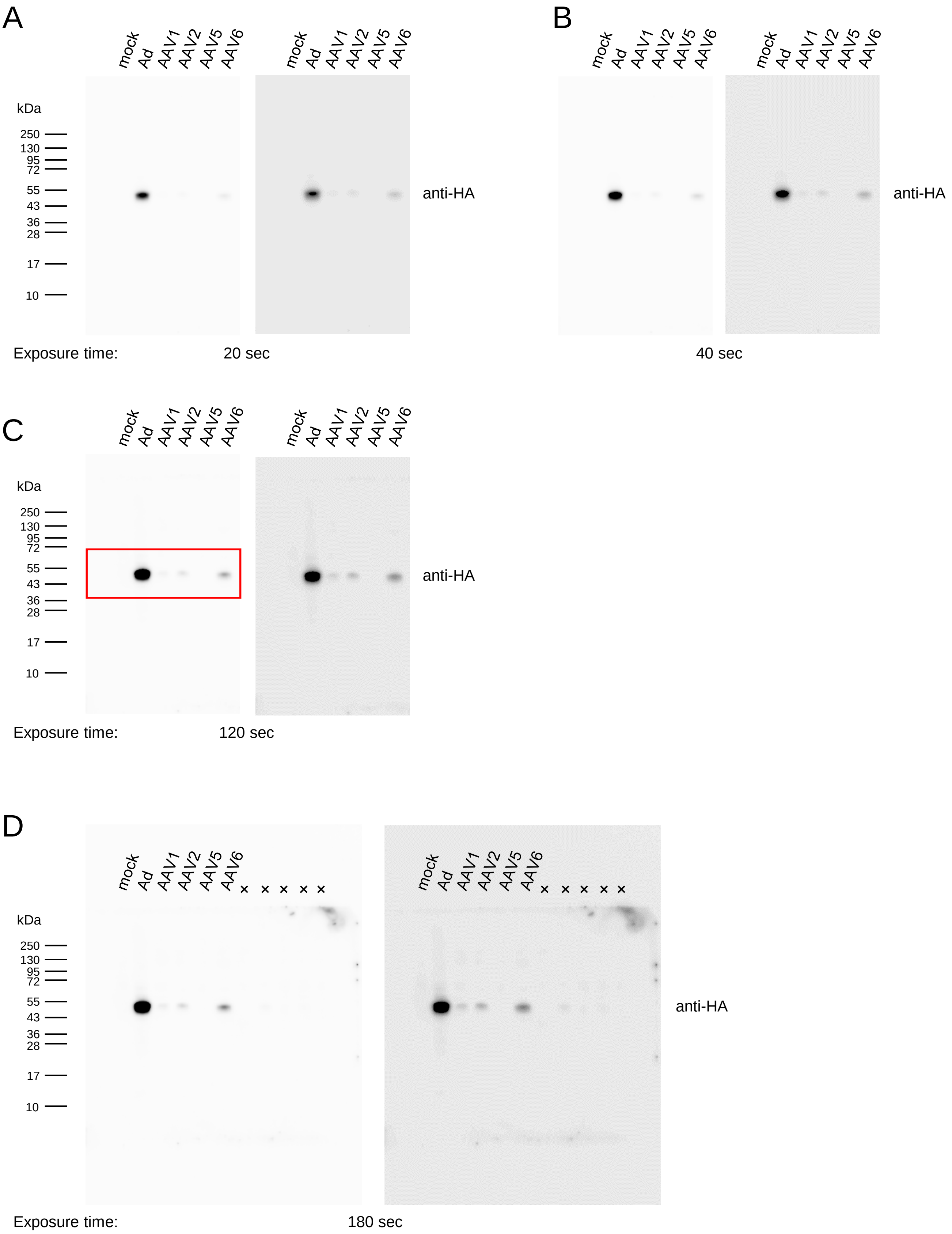
A
mock
Ad
AAV1
AAV2
AAV5
AAV6
kDa
250
130
95
72
55
43
36
28
17
10
anti-HA
Exposure time:
20 sec
B
mock
Ad
AAV1
AAV2
AAV5
AAV6
anti-HA
40 sec
C
mock
Ad
AAV1
AAV2
AAV5
AAV6
kDa
250
130
95
72
55
43
36
28
17
10
anti-HA
Exposure time:
120 sec
D
mock
Ad
AAV1
AAV2
AAV5
AAV6
× × × × ×
kDa
250
130
95
72
55
43
36
28
17
10
anti-HA
Exposure time:
180 sec

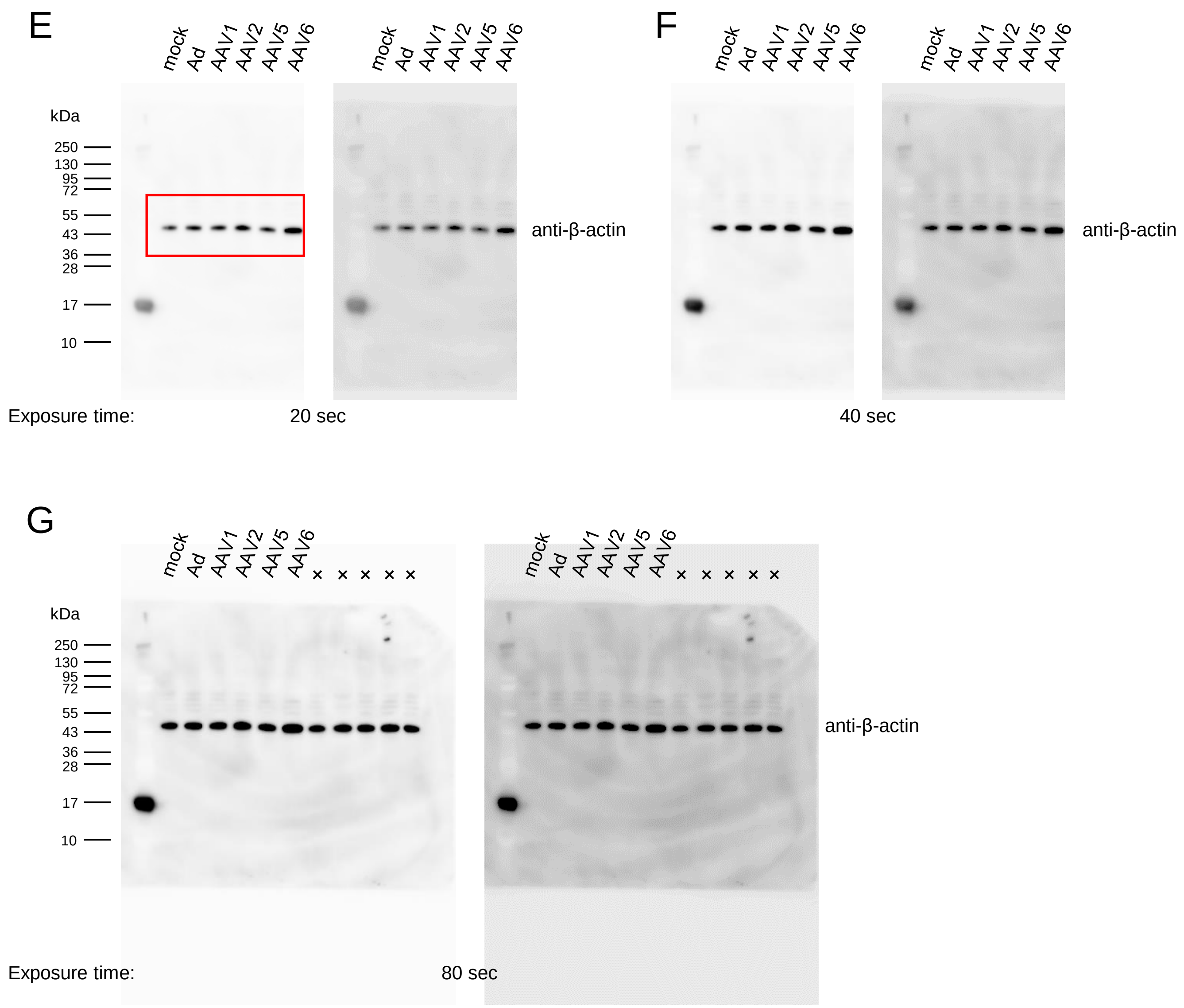

Supplementary Fig. S1. Expression levels of iCasp9 in immortalized NPCs transduced with the different viral vectors. Full-length images of the western blots showing the expression of iCasp9 with C terminal HA-tag in lysates of immortalized NPCs transduced with the viral vectors using an antibody against the HA-tag (A–D). β-actin expression levels were assessed in parallel as a loading control (E–G). The uncropped images are shown in D and G. The areas surrounded by the red line in C and E indicate the images shown in Fig. 1A. The images with reduced contrast are shown to confirm background noise (right panels).

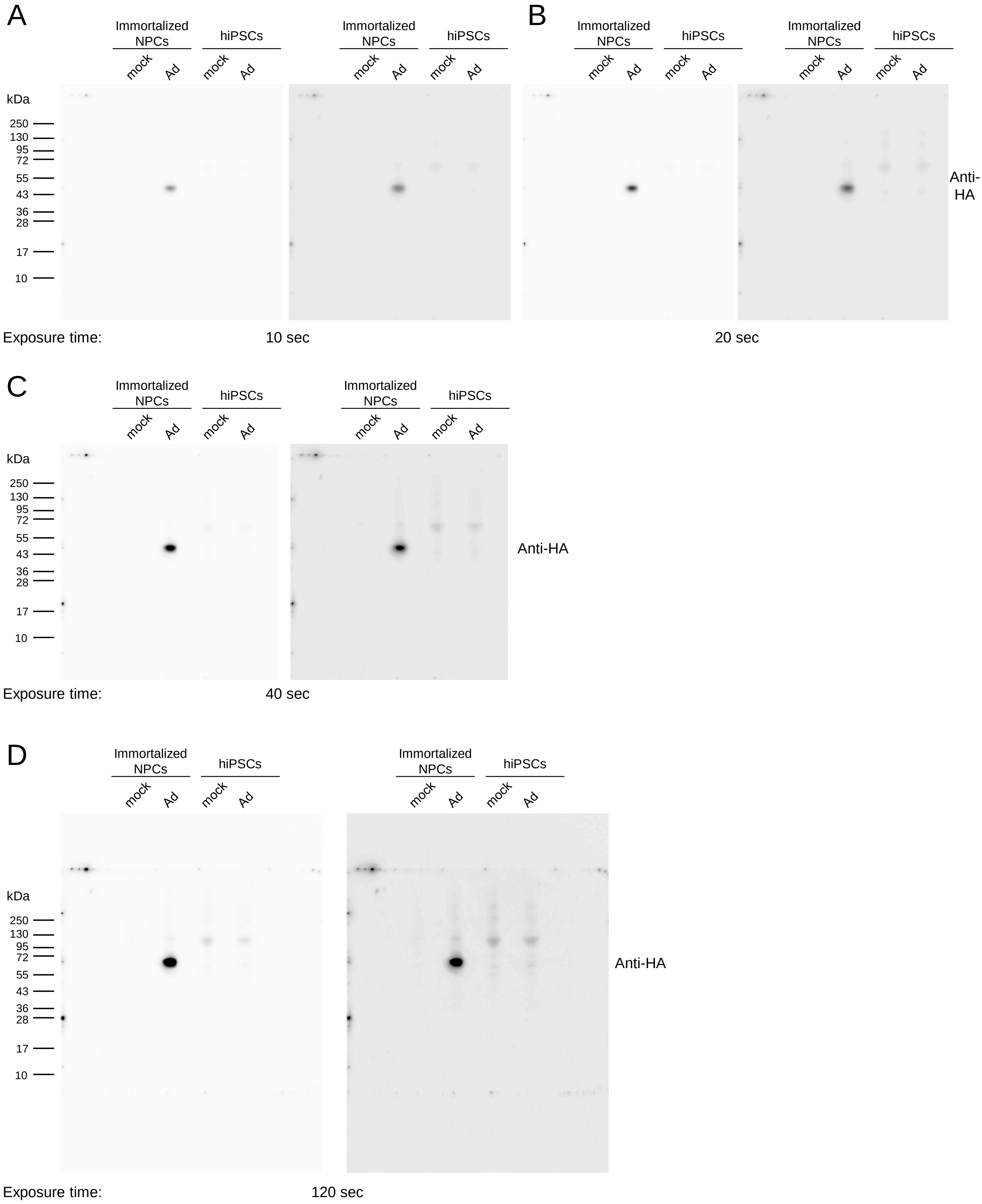
A
Immortalized NPCs
hiPSCs
mock
Ad
mock
Ad
kDa
250
130
95
72
55
43
36
28
17
10
Exposure time:
10 sec
B
Immortalized NPCs
hiPSCs
mock
Ad
mock
Ad
Anti-HA
20 sec
C
Immortalized NPCs
hiPSCs
mock
Ad
mock
Ad
kDa
250
130
95
72
55
43
36
28
17
10
Anti-HA
Exposure time:
40 sec
D
Immortalized NPCs
hiPSCs
mock
Ad
mock
Ad
kDa
250
130
95
72
55
43
36
28
17
10
Anti-HA
Exposure time:
120 sec

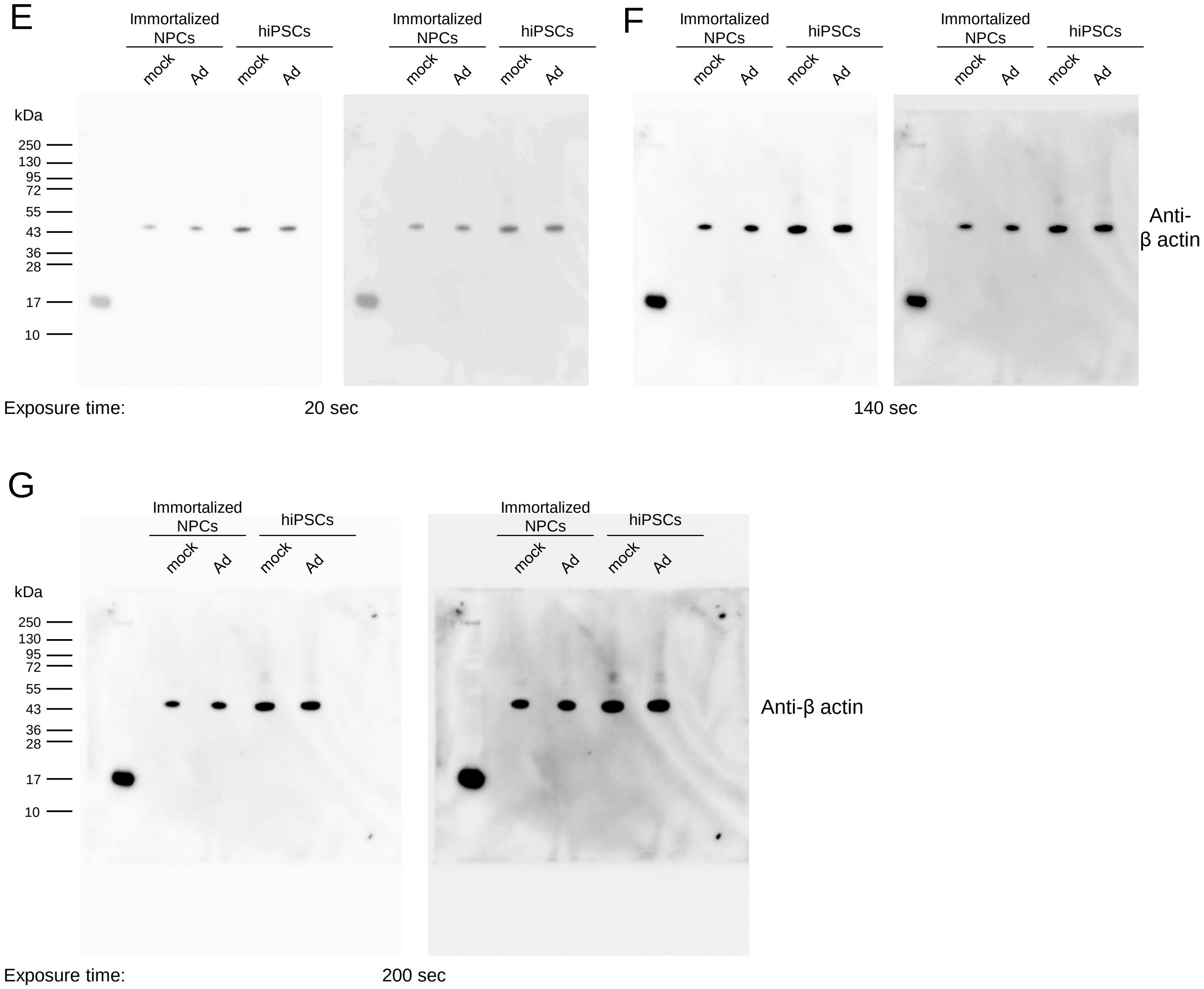

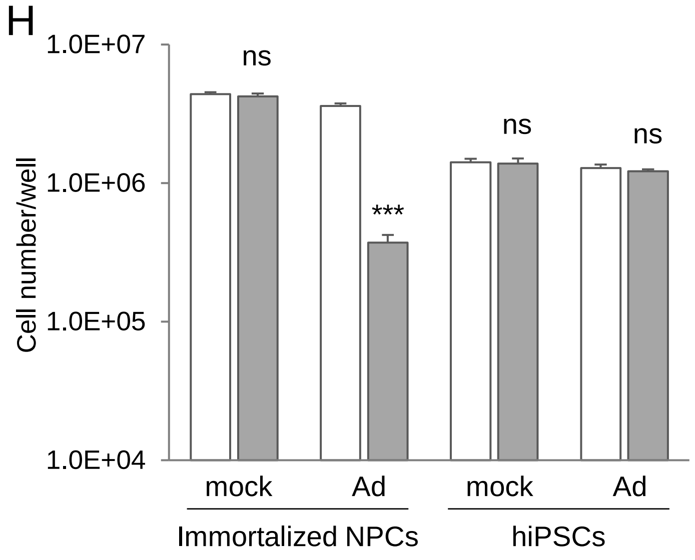

Supplementary Fig. S2. The Ad vector is strongly cytotoxic toward immortalized NPCs but not hiPSCs.

(A–G) The expression levels of iCasp9 with C terminal HA-tag in lysates of immortalized NPCs and hiPSCs transduced with the Ad vector (3 pfu/cell) were assessed by western blotting with an antibody against HA-tag (A–D). β-actin expression levels were assessed in parallel as a loading control (E–G). The uncropped images are shown in D and G. The images with reduced contrast are shown to confirm background noise (right panels). (H) The number of immortalized NPCs and hiPSCs transduced with the Ad vector (3 pfu/cell) was counted with and without AP1903 treatment (open column, without AP1903; filled column, with AP1903). Data are presented as means ± SD (n = 3, biological replicates). The statistical significance of differences between the cells treated with and without AP1903 was determined by two-way ANOVA and SNK post-hoc test (***$P < 0.005$; ns, not significant *vs.* without AP1903, SigmaPlot Version 12.5).

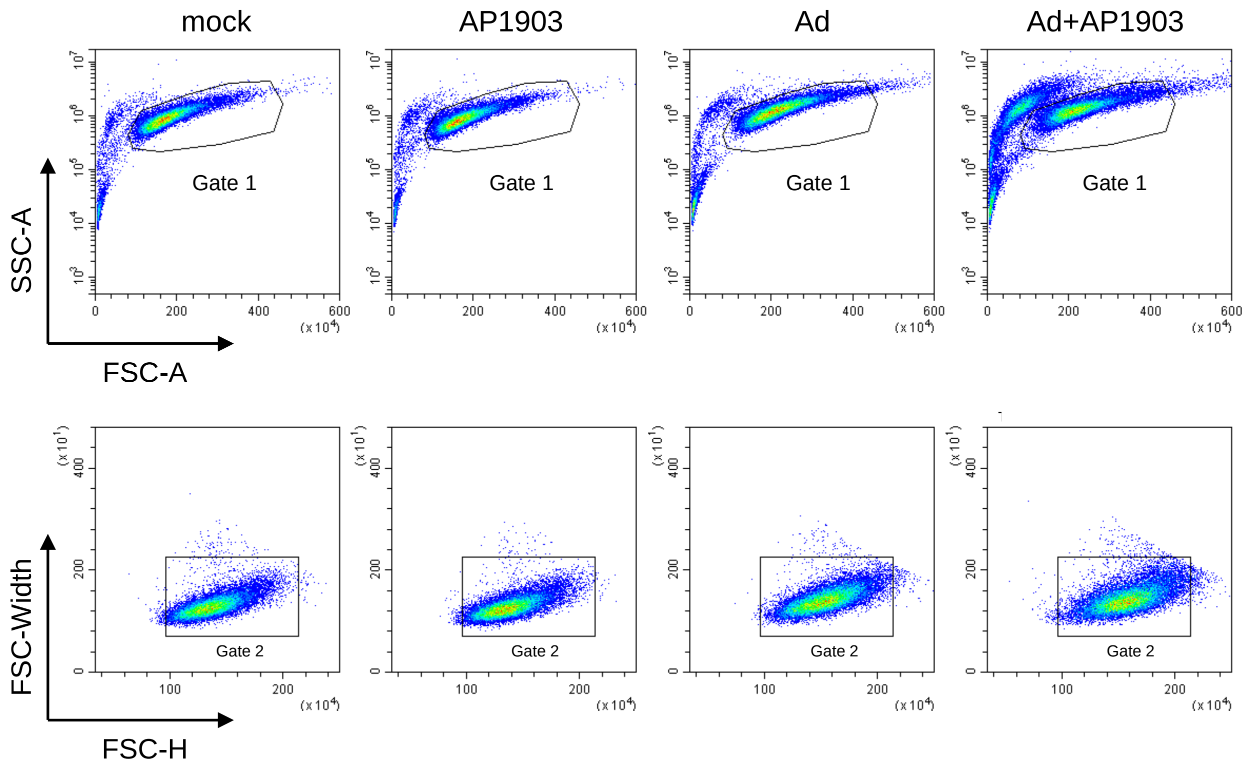

Supplementary Fig. S3. Pseudo color plots of FSC-A/SSC-A and FSC-H/FSC-Width in flow cytometry analysis shown in Fig. 2.

Representative pseudo color plots of the data from three independent experiments are shown. Cells in gates 1 and 2 were analyzed.

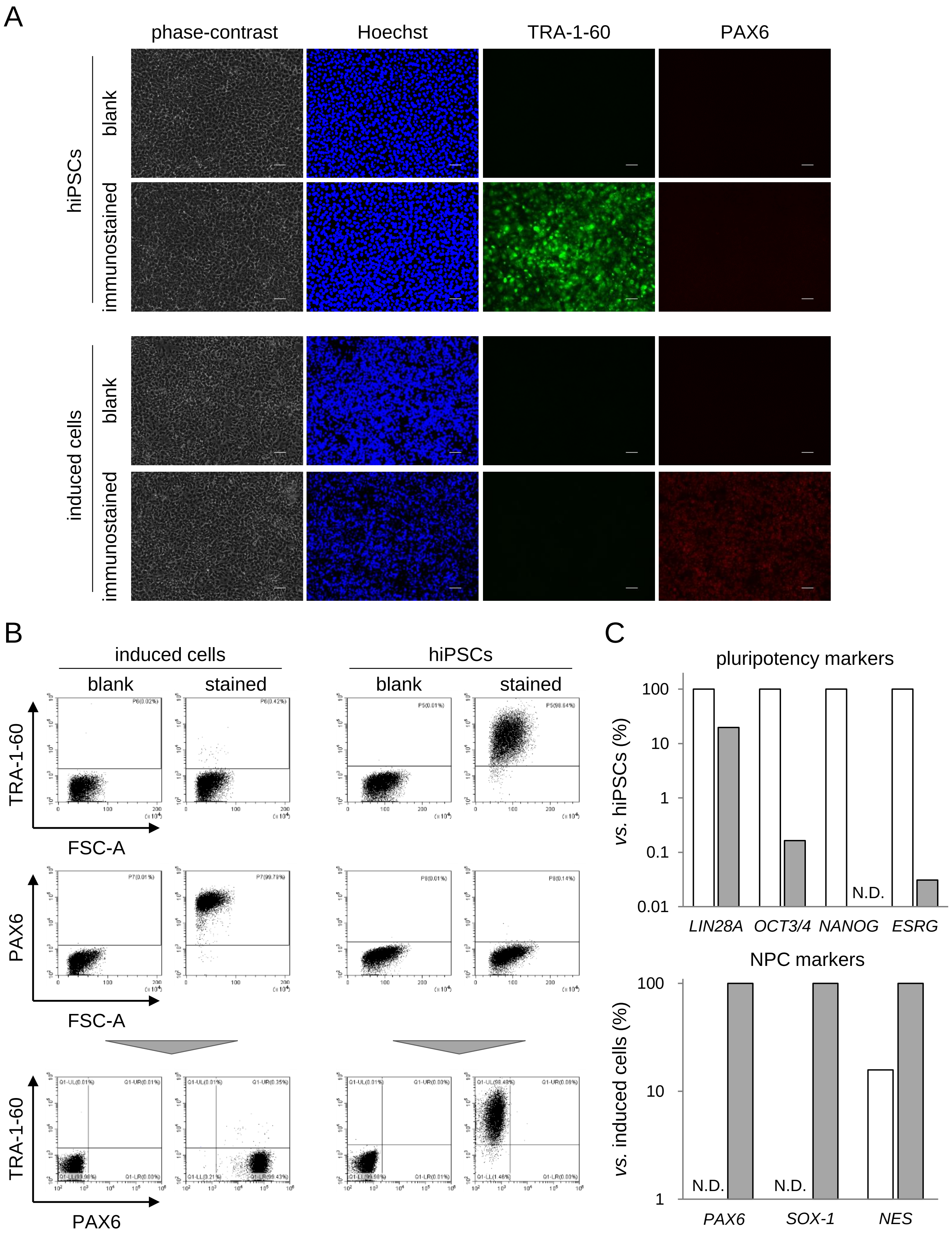

A
phase-contrast
Hoechst
TRA-1-60
PAX6
hiPSCs
blank
immunostained
induced cells
blank
immunostained
B
induced cells
hiPSCs
blank
stained
blank
stained
TRA-1-60
FSC-A
PAX6
FSC-A
TRA-1-60
PAX6
C
pluripotency markers
vs. hiPSCs (%)
100
10
1
0.1
0.01
N.D.
LIN28A
OCT3/4
NANOG
ESRG
NPC markers
vs. induced cells (%)
100
10
1
N.D.
N.D.
PAX6
SOX-1
NES

Supplementary Fig. S4. Differentiation of hiPSCs into NPCs.

The expression of TRA-1-60 and PAX6 in induced cells (hiPSC-NPC preparations) and hiPSCs was determined by immunostaining (A) and flow cytometry (B). The scale bar is 50 µm. (C) Quantitative real-time polymerase chain reaction analysis of pluripotent markers (*LIN28A*, *OCT3/4*, *NANOG*, and *ESRG*) and NPC markers (*PAX6*, *SOX-1*, and *NES*). Total RNA was isolated from induced cells and hiPSCs. The relative expression of target genes in 25 ng of total RNA was normalized to that of GAPDH (glyceraldehyde-3-phosphate dehydrogenase) transcript. (open column, hiPSCs; filled column, induced cells; N.D., not determined)

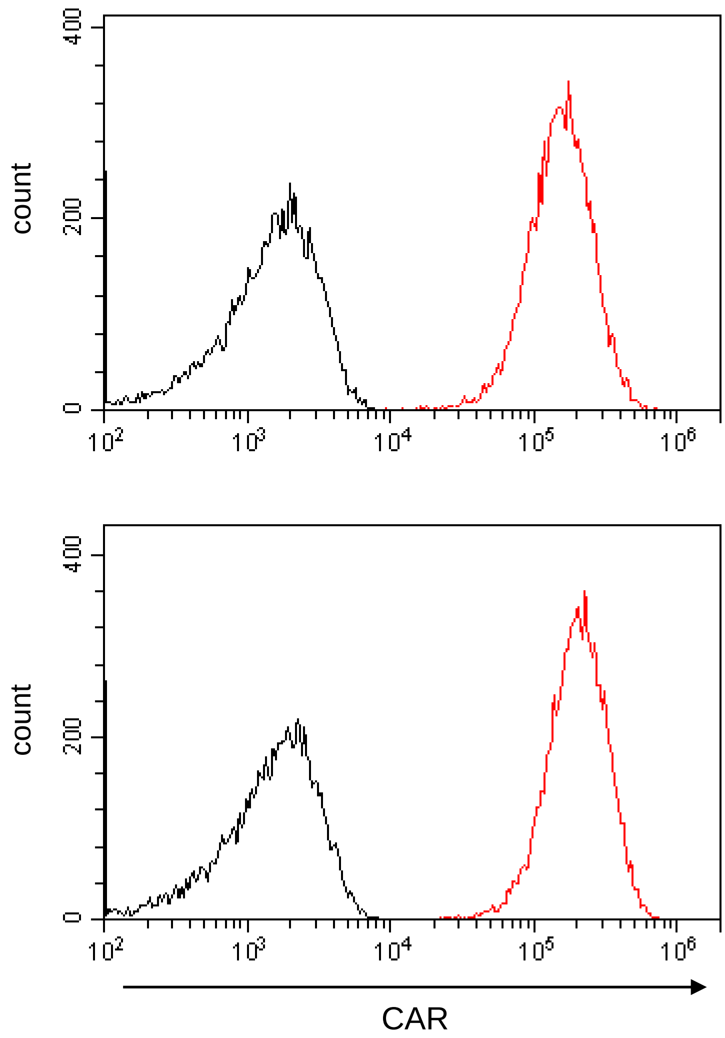

Supplementary Fig. S5. Expression levels of CAR on the cell surface of immortalized NPCs and induced cells (hiPSC-NPC preparations).

Immortalized NPCs (upper panel) and induced cells (hiPSC-NPC preparations, lower panel) were incubated with 2.5 µg/mL of an anti-CAR antibody (red lines) and a control antibody (black lines) on ice for 1 h. Subsequently, cells were washed twice with PBS and incubated with an allophycocyanin-labeled secondary antibody (1:2000) on ice for 1 h. After being washed thoroughly, the stained cells were analyzed using flow cytometry.

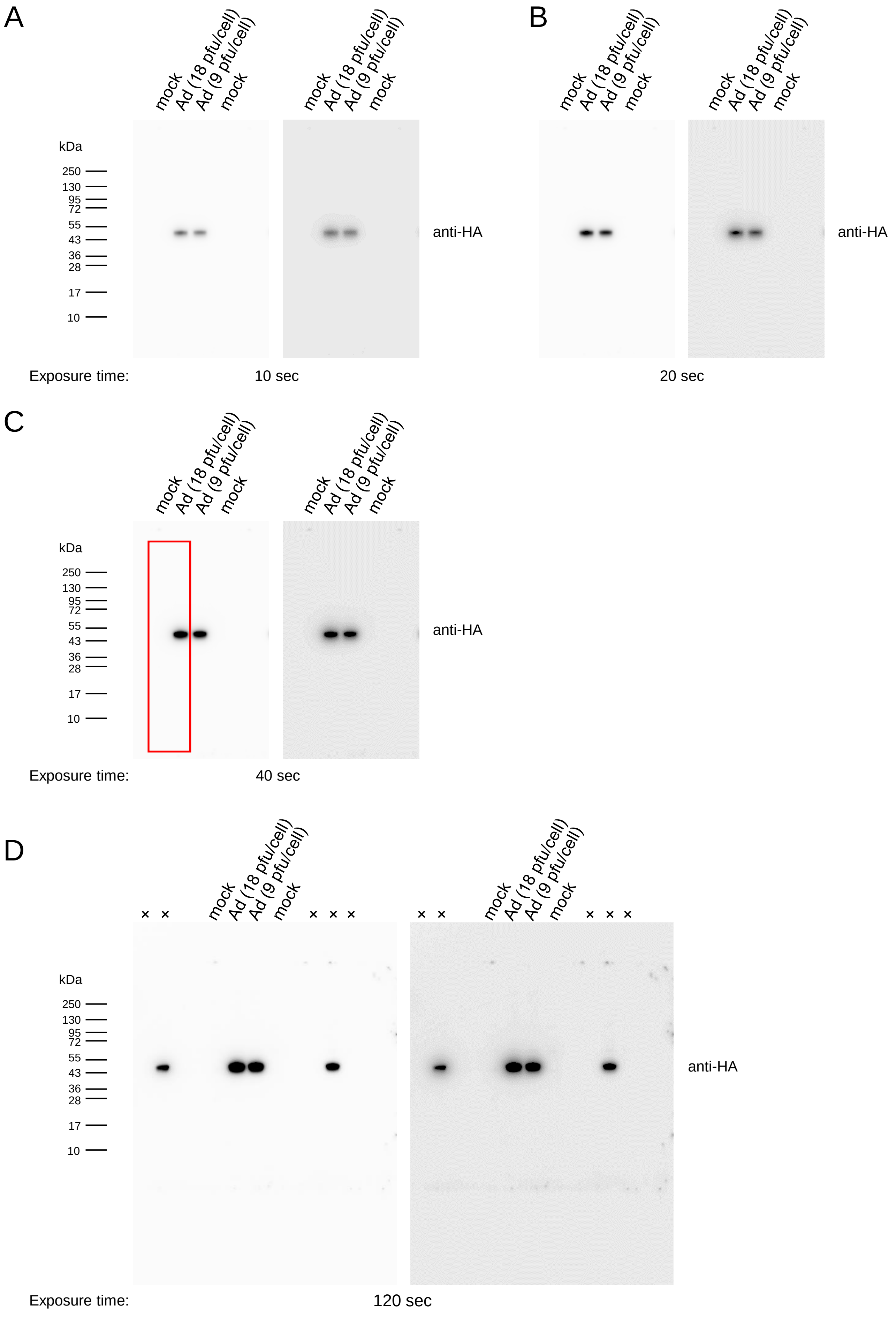
A
B
C
D
mock
Ad (18 pfu/cell)
Ad (9 pfu/cell)
mock
kDa
250
130
95
72
55
43
36
28
17
10
anti-HA
Exposure time:
10 sec
20 sec
40 sec
120 sec
×

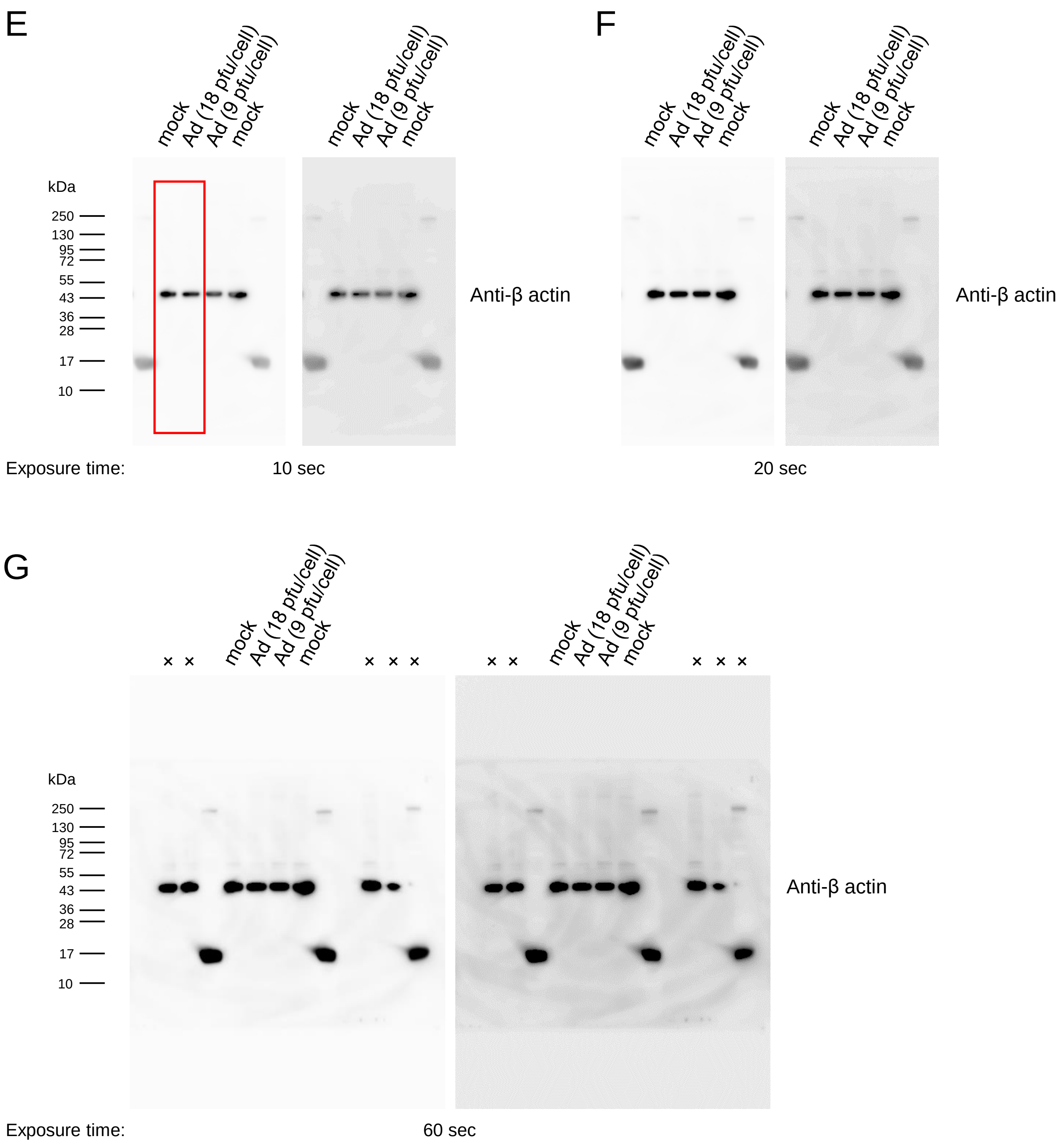

Supplementary Fig. S6. Expression levels of iCasp9 in hiPSC-NPC preparations transduced with the Ad vector. The expression levels of iCasp9 with C terminal HA-tag in lysates of hiPSC-NPC preparations transduced with the Ad vector (9 and 18 pfu/cell) were assessed by western blotting with an antibody against HA-tag (A–D). β-actin expression levels were assessed in parallel as a loading control (E–G). The uncropped images are shown in D and G. The areas surrounded by the red line in C and E indicate the images shown in Fig. 4A. The images with reduced contrast are shown to confirm background noise (right panels).

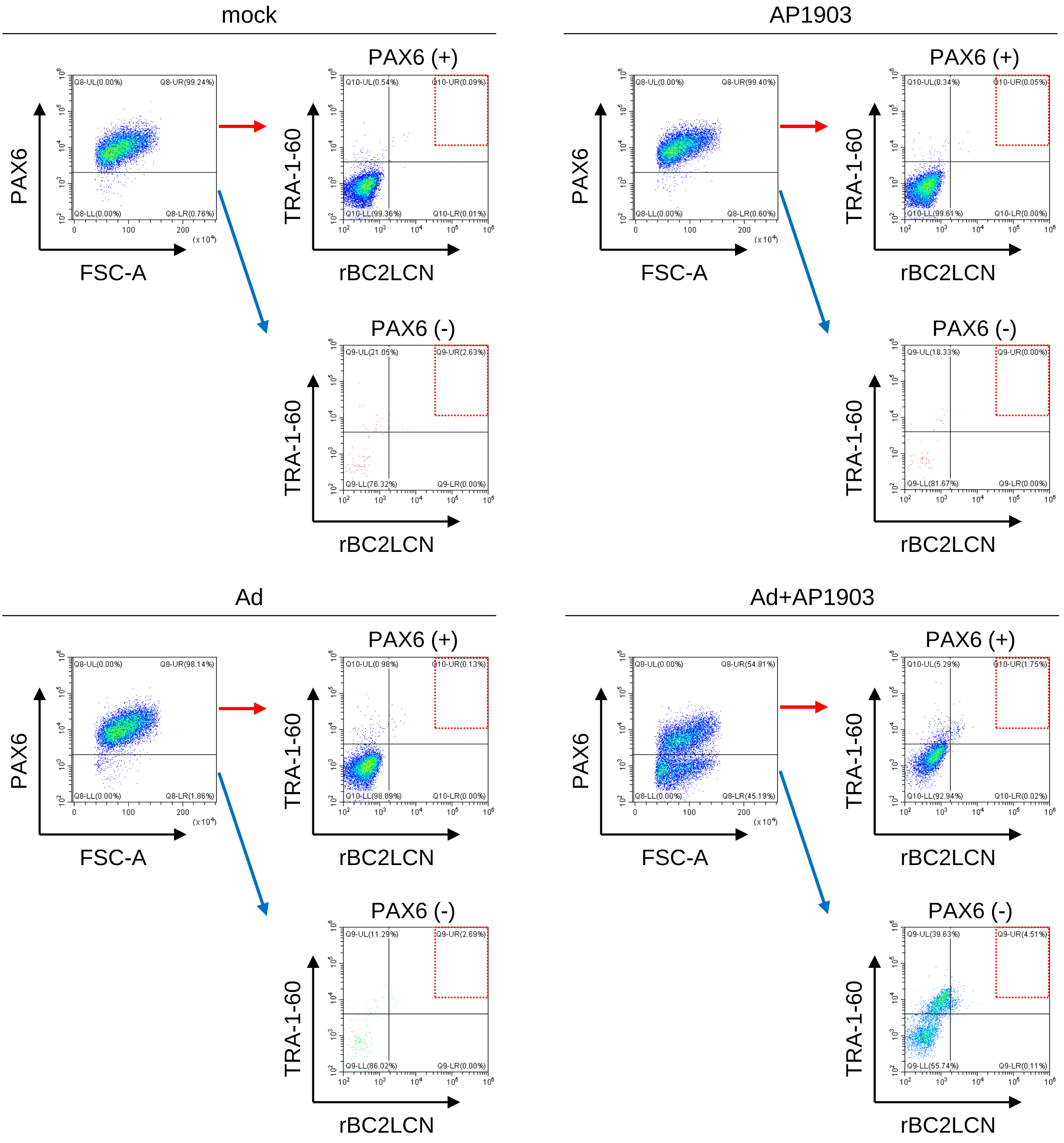

Supplementary Fig. S7. Flow cytometry analysis for hiPSC-NPC preparations treated with the Ad vector and AP1903. hiPSC-NPC preparations were transduced with the Ad vector at 18 pfu/cell for 24 h, followed by adding 10 nM AP1903. Twenty-four hours after treatment, cells were harvested by treatment with Accutase, and the cell suspension was incubated with an anti-TRA-1-60 monoclonal antibody conjugated with DyLight 650 (1:100) and with a FITC conjugated rBC2LCN (1:100) on ice for 1 h. The cells were fixed and permeabilized using a Foxp3/Transcription Factor Staining Buffer Set on ice for 30 min, followed by incubation with PE mouse anti-human Pax6 clone O18-1330 (1:40) on ice for 30 min. After being washed thoroughly, the stained cells were analyzed by flow cytometry. Representative pseudo color plots of the data from three independent experiments are shown. No cell was detected in the areas surrounded by the red dotted line (highly positive for both TRA-1-60 and rBC2LCN), which indicates the windows of hiPSCs in Figs. 5 and 6 for reference.

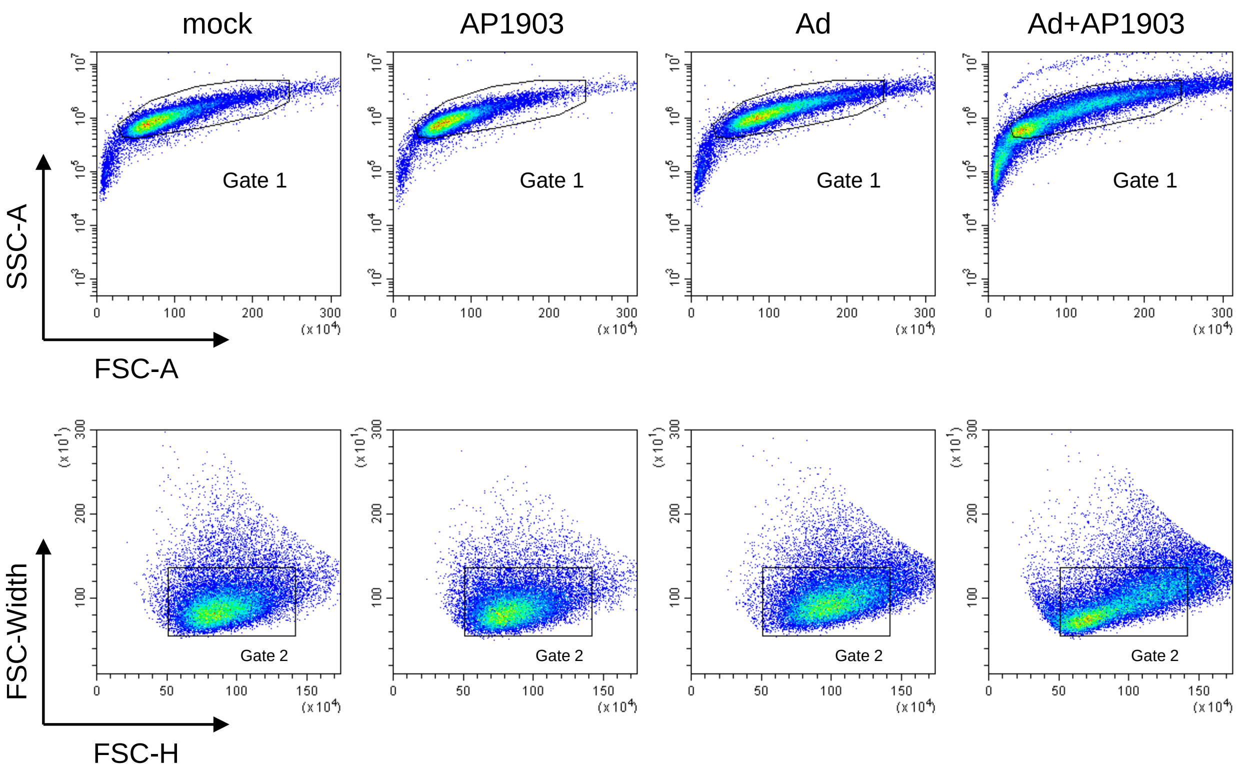

Supplementary Fig. S8. Pseudo color plots of FSC-A/SSC-A and FSC-H/FSC-Width in flow cytometry analysis shown in Fig. 5.

Representative pseudo color plots of the data from three independent experiments are shown. Cells in gates 1 and 2 were analyzed.

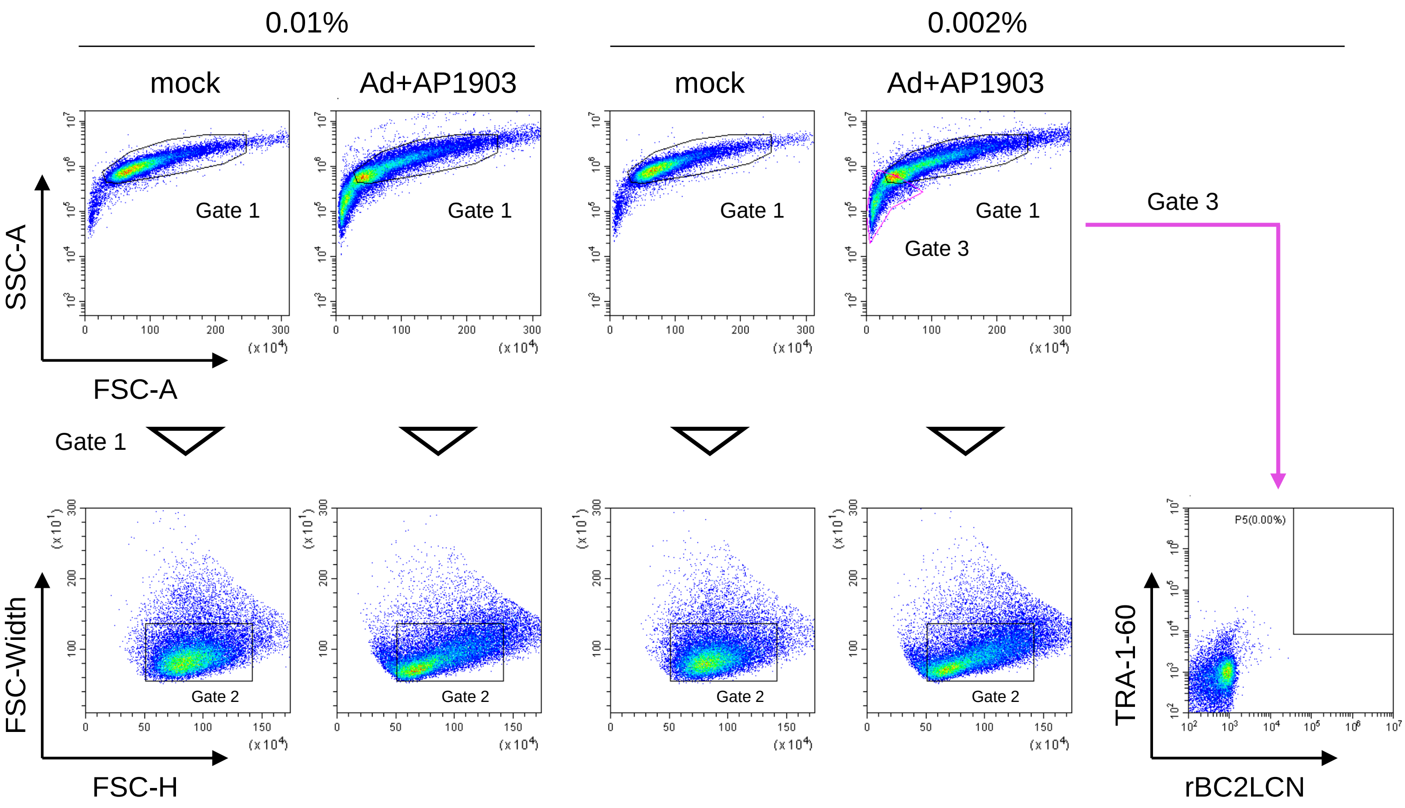

Supplementary Fig. S9. Pseudo color plots in flow cytometry analysis shown in Fig. 6.

Representative pseudo color plots of the data from three independent experiments are shown. Cells in gates 1 and 2 were analyzed and the results were shown in Fig. 6. For the 0.002% hiPSC group, the population of the estimated dead cells including in cells treated with the Ad vector and AP1903 was surrounded by gate 3 and was determined the expression of TRA-1-60 and rBC2LCN. No cell positive for TRA-1-60 and rBC2LCN was detected in three independent experiments.

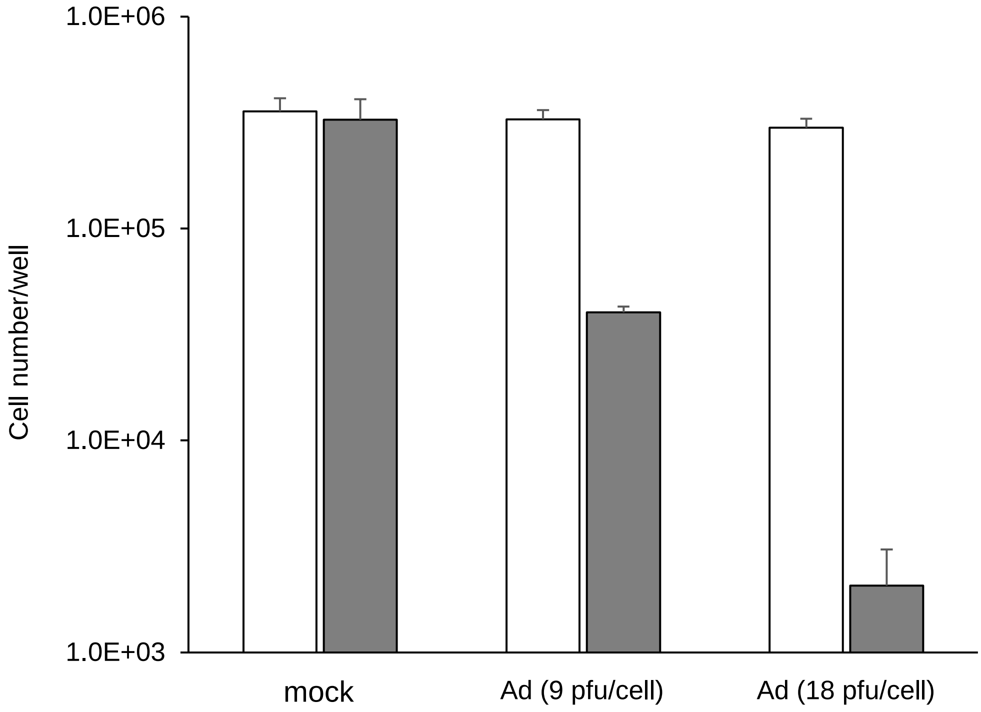

Supplementary Fig. S10. High titer of the Ad vector was needed to kill hiPSC-NPC preparations with high efficiency. The number of living cells in hiPSC-NPC preparations transduced with the Ad vector (9 and 18 pfu/cell) was counted after treatment with or without AP1903 (open column, without AP1903; filled column, with AP1903). Data are presented as means ± SD (n = 3, biological replicates).

Cell Type	Phenotype rBC2LCN	TRA-1-60	PAX-6	Ad (-) AP1903 (-)	*Fold Change*	Ad (-) AP1903 (+)	*Fold Change*	Ad (+) AP1903 (-)	*Fold Change*	Ad (+) AP1903 (+)	*Fold Change*
Immature non-NPC cells (other than hiPSCs)	+	+	-	0.013 ± 0.02	*1.0*	0.009 ± 0.01	*0.7*	0.033 ± 0.04	*2.55*	2.11 ± 0.42	*163.5*
	-	+	-	0.16 ± 0.10	*1.0*	0.082 ± 0.05	*0.5*	0.20 ± 0.15	*1.25*	14.95 ± 2.23	*95.5*
	+	-	-	0.003 ± 0.01	*1.0*	0.00 ± 0.00	*0.0*	0.00 ± 0.00	*0.0*	0.11 ± 0.08	*34.2*
		total		0.17 ± 0.10	*1.0*	0.09 ± 0.04	*0.5*	0.23 ± 0.14	*1.3*	17.17 ± 17.17	*99.4*
Immature cells partially differentiated into NPCs	+	+	+	0.063 ± 0.05	*1.0*	0.041 ± 0.01	*0.7*	0.11 ± 0.06	*1.7*	0.88 ± 0.33	*13.9*
	-	+	+	0.46 ± 0.27	*1.0*	0.33 ± 0.10	*0.7*	0.89 ± 0.09	*1.9*	2.11 ± 1.28	*4.6*
	+	-	+	0.007 ± 0.01	*1.0*	0.00 ± 0.00	*0.0*	0.00 ± 0.00	*0.0*	0.050 ± 0.05	*7.6*
		total		0.53 ± 0.32	*1.0*	0.37 ± 0.09	*0.7*	1.00 ± 0.12	*1.9*	3.04 ± 1.56	*5.7*
Differentiated non-NPC cells	-	-	-	0.50 ± 0.17	*1.0*	0.35 ± 0.09	*0.7*	1.09 ± 0.40	*2.2*	27.44 ± 8.17	*55.1*
NPCs	-	-	+	98.80 ± 0.28	*1.0*	99.19 ± 0.17	*1.0*	97.68 ± 0.56	*1.0*	52.35 ± 6.60	*0.5*

Supplementary Table S1. Proportions (%) of cell types surviving after the treatment with the Ad vector and AP1903.

The values except fold changes are presented as means ± SD of three independent experiments shown in supplementary Fig S6. Fold changes were calculated relative to cells not treated with the Ad vector and AP1903.

Gene	Probe sequences (5´ - 3´)	Forward primer sequences (5´- 3´)	Reverse primer sequences (5´- 3´)
LIN28	[FAM]-CGCATGGGGTTCGGCTTCCTGTCC-[TAMRA]	CACGGTGCGGGCATCTG	CCTTCCATGTGCAGCTTACTC
OCT3/4	[FAM]-CGGACCACATCCTTCTCGAGCCCAAGC-[TAMRA]	GAAACCCACACTGCAGCAGA	TCGCTTGCCCTTCTGGCG
NANOG	[FAM]-TGCTGAGGCCTTCTGCGTCACACC-[TAMRA]	CTCAGCTACAAACAGGTGAAGAC	TCCCTGGTGGTAGGAAGAGTAAA
ESRG	[FAM]-ACCACCAAACAGGCTTTGTGTGAGCAACA-[BHQ1]	GGACGCCGAGCTTTAGCC	TCCCTTTCATGCGCGTCTG
PAX6	[FAM]-CCAGCCAGACCTCCTCATACTCCTGCAT-[TAMRA]	GGCAAATAACCTGCCTATGCAAC	ACTCCGCCCATTCACCGAA
SOX-1	[FAM]-AGAAAACGCTTTCCGCTTCCTCCGTAGG-[TAMRA]	GACTGAACTTCGGTGTTTTCTTGA	GCCTCTCGCCTCGTTTTGAC
NES	[FAM]-TCTGTAGGCCCTGTTTCTCCTGCTCCA-[TAMRA]	GGCAGCGTTGGAACAGAGG	CCTTCCAGGACCTGAGCGA

Supplementary Table S2. Probe and primer sequences.
